# Substrate Specificity and Immunological Implications of *Cutibacterium acnes* Phage Endolysins

**DOI:** 10.4014/jmb.2509.09038

**Published:** 2026-01-21

**Authors:** Hafiza Hira Bashir, Muhammad Adeel Hasnain, Gi-Seong Moon

**Affiliations:** 1 Major in Biotechnology, Korea National University of Transportation, Jeungpyeong 27909, Republic of Korea; 2 Major in IT·Biohealth Convergence, Department of IT·Energy Convergence, Graduate School, Korea National University of Transportation, Chungju 27469, Republic of Korea; 3 4D Convergence Technology Institute, Korea National University of Transportation, Jeungpyeong 27909, Republic of Korea

**Keywords:** Acne vulgaris, Substrate selectivity, Bacteriophages, Genomics and proteomics, Molecular docking, Immuno-modulatory functions

## Abstract

*Cutibacterium acnes* resistance to antibiotics poses a significant challenge in treating acne vulgaris. Bacteriophages offer a promising alternative to overcome this challenge given their specificity. In the current study, 15 bacteriophages were isolated from acne affected volunteers and subjected to whole genome sequencing to characterize their genetic features, diversity and endolysins encoding genes for further structural and functional analysis. Five representative endolysins (CAP 1-1, 6-3, 7-1, 10-3, and 12-3) were chosen for structural and functional analysis after average nucleotide identity (ANI) analysis where 5 different endolysins were categorized. Furthermore, molecular docking studies assessed the binding affinities of endolysins to common peptidoglycan fragments of *C. acnes* cell wall, identifying variations in the binding interactions as CAP 6-3, 7-1, and 12-3 had greater affinities for the NAG–NAM dimer, while CAP 1-1 and CAP 10-3 interacted preferentially with NAM-L-alanyl-D-isoglutamine (MDP). Residue-level interaction mapping revealed several conserved histidine residues; ASP170 is conserved in peptide-targeting endolysins. These results imply that *C. acnes* phage (CAP) endolysins may be functionally differentiated into peptide-targeting and glycan-targeting classes based on their substrate-binding preferences, in addition to the traditional classification of endolysins by bond-cleaving activity. Notably, by interfering with NOD2-mediated signaling, MDP binding may increase the potential for modifying host immunological responses. Together, this research offers novel molecular understandings of the substrate selectivity and possible immunomodulatory functions of CAP phage endolysins. These results provide computational insights into substrate specificity and potential immunomodulatory mechanisms of *C. acnes* phage endolysins therefore experimental validation is necessary to verify their biological and therapeutic significance.

## Introduction

Acne vulgaris is one of the most common skin disorders, affecting roughly 9.4% of the world population [1] most commonly teenagers [2]. It can lead to social, emotional problems ranging from depression to suicidal thoughts [3, 4]. *Cutibacterium acnes*, the rod-shaped, aerotolerant anaerobic Gram-positive bacterium, plays a significant contributory role in pathophysiology of acnes [5]. Due to increasing instances of antibiotic-resistant strains, alternative antibacterial agents are required [6].

Bacteriophages are viruses that infect and kill bacteria through a multistep process characterized by host-specific receptor recognition and genome injection. A phage first recognizes and binds to its bacterial host via receptor-binding proteins, then ejects its genome into the cytoplasm through a channel formed by the tape measure protein. Once inside, lytic phages hijack the bacterial machinery to replicate their genomes and produce structural and lysis proteins, ultimately causing host cell lysis and release of new phage particles [7].

Bacteriophage therapy, *i.e.* therapeutic use of bacteriophages, has been under consideration since their discovery as a potential antibacterial strategy, before the arrival of the antibiotic era. The concerns related to the therapeutic use of phages together with the widespread success of antibiotics, pushed this concept into a shade, and its application remained largely limited to the Soviet Union [8]. However, the exacerbating situation of antibiotic resistance has renewed interest in phage therapy, especially to treat life-threatening infections caused by multidrug-resistant bacteria [9]. Recent research has shown that phages can be used to replace or augment antibiotic treatment, particularly against multidrug-resistant bacterial infections [10].

Although phages present several advantages over the traditional antibiotics like specificity and reduced side effects, there are still challenges associated with the use of phages *e.g.* possibility of horizontal gene transfer of antibiotic resistance genes (ARGs) and reduced activity due to immune response [11]. Therefore, lytic phage proteins endolysins are also being explored because of their additional benefits like fast bactericidal activity, non-proliferation and low possibility of resistance (as these attack vital and highly conserved bonds within the peptidoglycan) [12].

Endolysins are important proteins with reference to the last stage of lytic cycle of bacteriophages. Endolysins degrade the target cell wall by binding to peptidoglycan and hydrolyzing bonds within peptidoglycan after it passes through the pores in the cell membrane created by holins [13, 14]. Although the composition of peptide subunit varies and the structure of interpeptide bridges varies, the carbohydrate backbone consisting of N-acetylglucosamine (NAG) and N-acetylmuramic acid (NAM) remains the common feature among all bacteria [15] . Endolysins specific to Gram-positive bacteria including *C. acnes* usually consists of an enzymatically active N-terminal domain and cell wall binding C-terminal domain (CBD). Since experiments on the co-crystallization of specific parts of bacterial cell wall along with the endolysins don’t always lead to interpretable results, alternative approaches to explore mechanism of bacterial peptidoglycan and its components binding by endolysins should be utilized. Among these approaches molecular docking has emerged as an important tool in various fields to gain insights into protein-ligand interactions [16]. Currently there exists various academic and commercial programs to study protein-ligand interactions. Among these, Autodock Vina, which implies knowledge-based scoring function, offers high prediction accuracy along with short simulation time [17].

In this study, we investigated representative endolysins from *C. acnes* phages to elucidate their structural and functional characteristics. Sequence clustering and molecular docking revealed two functional classes of endolysins under study: peptide-targeting and glycan-targeting, distinguished by their binding preferences for muramyl dipeptide (MDP) or N-acetylglucosamine-N-acetylmuramic acid (NAG-NAM) dimers. Residue-level mapping highlighted conserved histidine residues, including unique ASP170, in peptide-targeting enzymes.

## Materials and Methods

### Bacterial Strains and Bacteriophage Isolation

This study used the *C. acnes* KCTC 3314 and KCTC 3320 strains, which were acquired from the Korean Collection for Type Cultures (KCTC, Republic of Korea). Reinforced Clostridial Medium (RCM) (BD, USA) was used to cultivate the strain in an anaerobic chamber (DG250; Don Whitley Scientific Ltd., UK) at 37°C.

Fifteen plaque forming bacteriophages against *C. acnes* were isolated from human skin acne lesions of volunteers as described previously [18]. The collection of acne lesion swabs was conducted under approval by the Korea National University of Transporatation-Institutional Review Board, IRB No. KNUT-IRB2020-19. Written informed consent was obtained from all volunteer participants prior to sample collection. Briefly, to isolate bacteriophages from the skin of volunteers having acne, sterile cotton swabs were swapped twice on the acne-affected area and put into RCM broth inoculated with 1 × 10^5^ CFU/ml of *C. acnes* KCTC 3314 or KCTC 3320 strain and cultured anaerobically at 37°C for 72 h. The cultured broth was then centrifuged at 11,000 × *g* for 10 min at 4°C, and the supernatant was recovered and filtered with 0.45 μm syringe filter (Anylab Co., Republic of Korea). Phage activity was analyzed by adding the 50 ul of diluted supernatant to 6 ml of RCM soft agar (1.2% agar, *w/v*) containing approximately 1 × 10^5^ CFU/ml of *C. acnes* KCTC 3314 or KCTC 3320, overlaid on an RCM agar plate and was incubated anaerobically at 37°C for upto 48 h. Single plaques were then picked up and added to the RCM broth inoculated with the target strain and incubated anaerobically at 37°C for up to 72 h. The culture was then centrifuged (11,000 × *g*,10 min, 4°C) and the supernatant was recovered, decimally diluted and loaded on RCM agar plates inoculated with the target strain for the enumeration of plaques.

### Genomic Characterization of Bacteriophages

The genomic DNA of phages was isolated using a phage DNA isolation kit (Norgen Biotek Co., Canada). The isolated genomic DNA were subjected to whole genome sequencing (WGS) on iSeq 100 sequencer (Illumina, Inc., USA) to obtain comprehensive genomic data following the protocols as previously described [19].

Following WGS, FastQC was used to ensure quality of high-throughput sequence data. Trimmomatic [20] was then used to improve the data quality for downstream analysis. MEGAHIT v1.2.9 [21] assembler was used to reconstruct genomes from short-read sequencing data. Assembly statistics were obtained from QUAST [22] and BUSCO [23] was used to check the percentage of genome completeness. BLASTn was used to check the degree of similarity with the previously known *C. acnes* bacteriophages.

The assembled genomic sequences were subsequently annotated using web-based tool PHASTER (https://phaster.ca/). From these annotations, the sequences of the endolysin proteins were extracted and each sequence was verified using BlastP. Further, the therapeutic potential of the phages was evaluated using the PhageLeads platform (https://phageleads.dk/). Identification of potential genes encoding tRNA was carried out using tRNAscan-SE v.2.0. [24]. Average Nucleotide Identity (ANI) was calculated using Geneious Prime 2025.0.

### Selection Criteria for Representative Endolysins

After ANI-based clustering, the five representative endolysins were selected based on (i) determining the maximum sequence divergence within each cluster using pairwise sequence identity, and (ii) ensuring that at least one endolysin represented each major ANI cluster. This approach avoided redundancy by capturing both inter-cluster and intra-cluster variability.

### Protein Structure Modeling and Ligand Selection

**Fig. 1:**
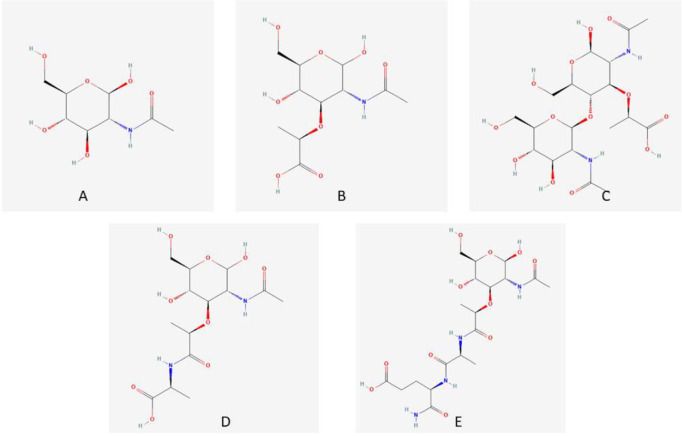
Structures for the ligands: NAG (A), NAM (B), NAG-NAM dimer (C), NAM-L-alanine (D) and NAM-L-alanyl-D-isoglutamine (E).

The extracted sequences of endolysin protein from five selected phages based on ANI were modelled using AlphaFold server (https://alphafoldserver.com/, accessed on 12, September 2025) and their PDB structures were downloaded. Moreover, the three-dimensional PDB structures of peptidoglycan fragments that are typical of various *C. acnes* species were obtained from PubChem database (https://pubchem.ncbi.nlm.nih.gov/). The list of the ligands used in the current study are N-acetylglucosamine (NAG, PubChem CID 24139) (Fig. 1A), N-acetylmuramic acid (NAM, PubChem CID 5462244) (Fig. 1B), NAG-NAM dimer (PubChem CID 72210857) (Fig. 1C), NAM-L-alanine (PubChem CID 10970945) (Fig. 1D), and NAM-L-alanyl-D-isoglutamine (PubChem CID 451714) (Fig. 1E). Finally, we converted PDB files to PDBQT format using the AutoDockTools v1.5.7 to use the aforementioned structures as ligand structures in Autodock Vina.

### Molecular Docking of Endolysins with Peptidoglycan Fragments Typical of *C. acnes*

**Fig. 2:**
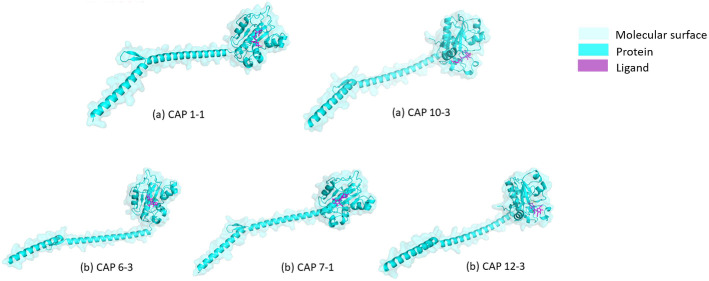
Surface views of the five representative *C. acnes* phage endolysins. CAP 1-1(a) and CAP 10-3(a) are shown with NAM–L-alanyl–D-isoglutamine (MDP), while CAP 6-3(b), CAP 7-1(b), and CAP 12-3(b) are shown with the NAG–NAM dimer, reflecting their respective best-binding ligands. Protein structures are shown in cyan (cartoon) with light cyan molecular surface, while ligands are displayed in magenta. Color legend: cyan (protein), light cyan (surface), magenta (ligand). Structural models are not drawn to scale; representations are intended for qualitative visualization of domain architecture and ligand positioning.

To assess the binding of endolysins from different bacteriophages with the typical peptidoglycan fragments as ligands, molecular docking was performed using AutoDock Vina. Because the active or catalytic sites of the *C. acnes* phage endolysins have not been experimentally determined in previous literature, we performed blind docking using AutoDock Vina default setting [25]. The docking grid box was defined to cover the entire protein structure, ensuring unbiased exploration of all potential ligand-binding cavities. For each protein, the grid center was automatically set to the geometric center of the model, and the grid box dimensions were expanded to fully enclose the protein’s bounding box. Docking was performed with an exhaustiveness value of 8, applying standard docking protocols and parameters to obtain protein docking complexes. This resulted in the generation of nine different poses of the ligand binding to each protein in pdbqt format, each with varying binding affinities. The top poses with the best binding affinities were then selected to generate the protein ligand complexes. The complexes were visualized using PyMol [26] to get surface view figures highlighting the interaction of ligand with proteins from different bacteriophages (Fig. 2).

### 2D Interaction Analysis of Endolysin-Ligand Complexes

The 2D interaction analysis of protein-ligand complexes were performed using Discovery Studio Visualizer (BIOVIA, USA). The complexes in pdb format were imported, and 2D illustrations were generated to highlight the interactions between protein residues and the ligand. Additionally, the domains were visualized using PyMol to identify the residues present in specific domains.

## Results

### Whole Genome Sequence (WGS) Analysis

**Fig. 3:**
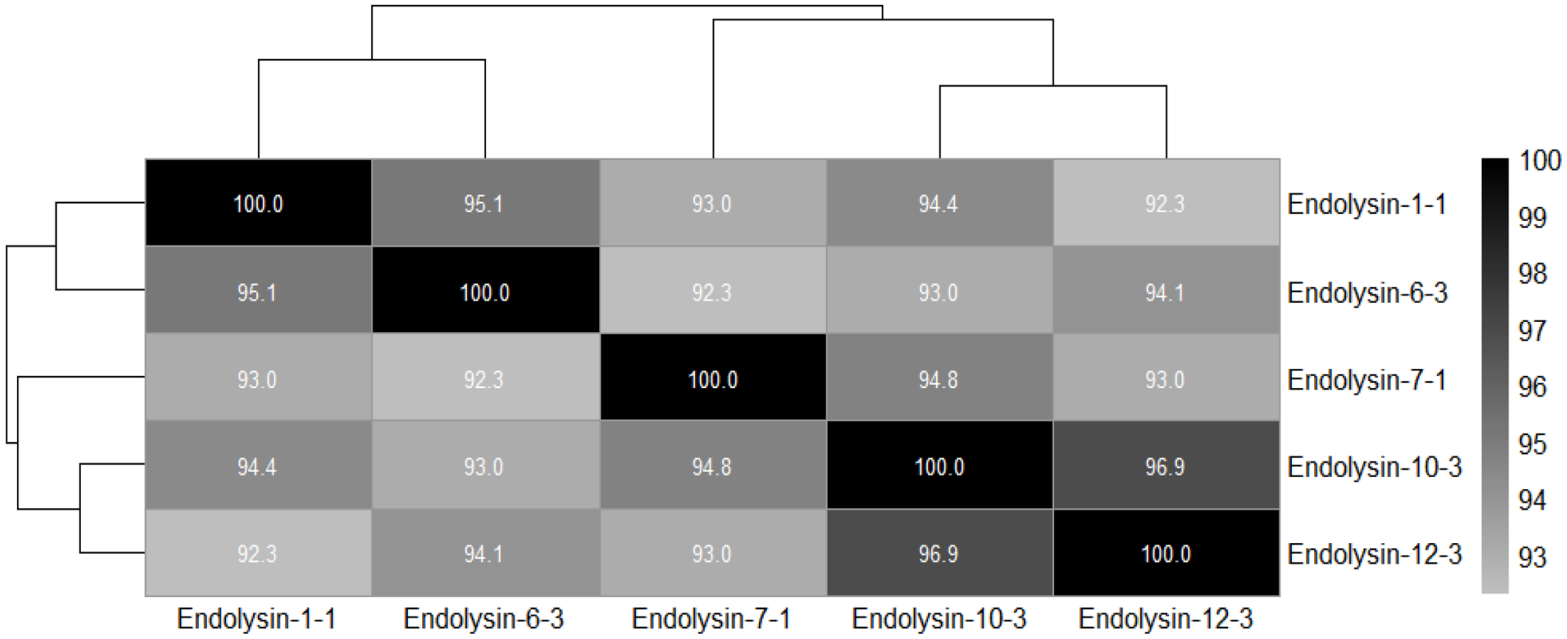
Average nucleotide identity of CAP endolysins.

During the WGS analysis, the assembly resulted in the total length of base pairs, comprising single number of contig, with N50 values. BUSCO analysis revealed the percentage of complete genes, indicating a high level of completeness. The 15 CAP genomes ranged in size from 29,449 to 29,983 bp, with 40 to 47 predicted protein-coding genes. Similarly, GC content of the sequenced phages showed a little variation from 53.59% to 54.25%. The sequences of isolated phages were submitted to GenBank and the accession numbers are provided in supplementary data (Table S1). Key genetic characteristics of the phages are provided in Table 1. Analysis of the nucleotide sequence similarity using BLASTn revealed that these phage genomes show a high degree of similarity not only among themselves but also with previously sequenced genomes of *C. acnes* phages. ANI analysis grouped the endolysins into three clusters of identical sequences and two different endolysins (1-1/1-2/1-3/2-1/2-2/10-1/12-1; 7-1/7-2/7-3/9-2; 12-2/12-3; 6-3; 10-3). To capture the observed diversity (92–100% sequence similarity across all clusters), five representative endolysins—CAP 1-1, 6-3, 7-1, 10-3, and 12-3—were selected for subsequent molecular docking analyses (Fig. 3).

The results of PhageLeads tool affirmed that all the phages exhibit a lytic lifestyle. None of the characterized bacteriophages possessed antibiotic resistance genes, virulence factors, and lysogeny-associated genes. Additionally, tRNAscan-SE results revealed the absence of tRNA-encoding genes in all the phages.

### Molecular Docking of Endolysins with Different Peptidoglycan Fragments

Molecular docking analysis revealed that endolysins from five bacteriophages exhibited varying binding affinities toward different peptidoglycan fragments of the *C. acnes* cell wall. The surface view of the five representative endolysins in complex with their highest-affinity ligands is shown in Fig. 2. CAP 1-1 and CAP 10-3 showed the greatest affinity for NAM-L-alanyl-D-isoglutamine (MDP), whereas CAP 6-3, 7-1, and 12-3 bound more strongly to the NAG–NAM dimer (Table 2). These findings suggest that the endolysins recognize specific structural features of *C. acnes* peptidoglycan, such as peptide- or glycan-containing regions, likely due to differences in their binding pocket residues.

### 2D Interactions Analysis

**Fig. 4:**
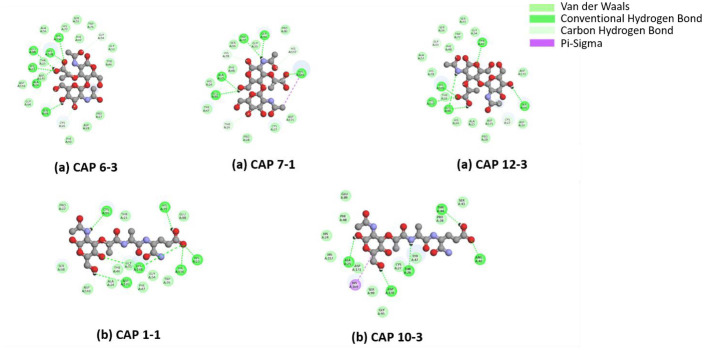
2D interaction analysis of endolysin from bacteriophages CAP 6-3(a), CAP 7-1(a), and CAP 12-3(a) with NAG-NAM dimer and endolysins from bacteriophages CAP 1-1(b) and CAP 10-3(b) with NAM-L-alanyl-D-isoglutamine, highlighting variations in binding patterns among the endolysins.

Key interaction residues and varying ligand binding preferences were identified by docking studies of the CAP endolysisns (Fig. 4). With NAM-L-alanyl-D-isoglutamine, CAP 1-1 and CAP 10-3 interacted preferentially, establishing hydrogen bonds with residues such CYS26, HIS23, HIS77, HIS156, HIS168, and ASP170 (CAP 1-1) and THR26, THR44, ALA25, ASP170, and ARG40 (CAP 10-3). The residues that interacted with NAG-NAM dimers in CAP 6-3, CAP 7-1, and CAP 12-3, on the other hand, were HIS23, HIS156, HIS168, GLU88, ALA24, SER98 (CAP 6-3); TRP77, GLY54, ALA25, GLU89, HIS169 (CAP 7-1); and TYR47, GLU89, SER99, HIS157, and HIS169 (CAP 12-3) (Table 3). A number of histidine and glutamate residues were notably found to be repeated in numerous CAP proteins, suggesting that they may play a significant role in ligand binding and recognition. A conserved mechanism for the NAM-L-alanyl-D-isoglutamine interaction is further suggested by the shared occurrence of ASP170 in CAP 1-1 and CAP 10-3, although unique residues in CAP 6-3, 7-1, and 12-3 most likely aid in the NAG-NAM dimer's selective binding. These findings demonstrate the conserved and distinctive characteristics of CAP protein-ligand interactions, which could be the basis for their varying substrate specificity.

## Discussion

*C. acnes* plays a significant role in the pathophysiology of acne vulgaris, although its mechanism remains poorly understood [27]. Frequent detection of antibiotic-resistant strains of *C. acnes* (especially the multidrug resistant strains) demands the dire need of alternative antimicrobials [28]. Phages, especially their lytic proteins namely the endolysins, have the potential to become an attractive alternate antimicrobial option considering the advantages they offer [29].

Regarding the quality of sequencing, the QUAST results showed the high N50 value and single contigs, indicative of a well-assembled genome with minimal fragmentation. The BUSCO completeness score further confirms the assembly’s quality, showing a high percentage of complete genes.

Genomes of the *C. acnes* bacteriophages show limited genetic diversity as opposed to the phages from other bacterial species. Even *C. acnes* phage separated by geographical regions or long span of time show high similarity [30]. In agreement with the previous observations, the phages investigated in this study showed a high degree of nucleotide similarity (>85%) among themselves and with the previously reported *C. acnes* phages. Additionally, the extent of variation in total genome size, percentage GC content and number of protein coding sequences was quite limited. All these observations align with the previous reports describing similar genomic size, little-to-no variation in GC content and similar number of protein coding regions. These findings show quite a different nature of the *C. acnes* phage as phages from the other species differ from these phages in almost all aspects. Sørensen *et al*., for example, reported a significant variation in genome sizes and GC content in the coliphages [31].

It has been observed that phages harbor GC content that is correlated with that of their host. *C. acnes* phages, however, does not seem to follow this observation as their GC content is not only lacking diversity, but it also does not overlap with that of their host bacterium [32]. Different plausible explanations have been put forward regarding this observation, for example anti-*C. acnes* phages may have recently adapted for growth in their host [30]. The limited diversity may also be linked with the fact that their host strain is predominantly found in pilosebaceous region of the human body, thus these phages are constrained from having interactions with phages from other species.

The low genomic and structural diversity observed among *C. acnes* phages may also have implications for phage therapy. On one hand, a high degree of functional stability is suggested by this conservation, which could be beneficial for developing consistent therapeutic formulations. However, limited genomic diversity could reduce the availability of phages needed to combat emerging bacterial resistance [30]. A limited variety of receptor-binding proteins and lytic domains may limit adaptation against *C. acnes* strains that develop phage-resistance mechanisms [33]. As a result, while the conserved nature of these phages supports their potential therapeutic utility, it also emphasizes the importance of engineered or synthetic diversification strategies to widen host range and increase long-term efficacy.

The present study investigated the substrate specificity of the endolysins of 15 bacteriophages that infect *C. acnes*. Whole genome sequencing demonstrated the potential safety of isolated phages for therapeutic uses by confirming that they all have lytic lifecycles and are free of virulence, antibiotic resistance, and lysogeny-associated genes.

Additionally, ANI analysis identified three endolysin sequence clusters and two different endolysins, suggesting a high level of phage conservation. This also enabled the selection of five representative endolysins (CAP 1-1, 6-3, 7-1, 10-3, and 12-3) for in-depth molecular interaction investigations.

The molecular docking and 2D interaction analysis revealed the unique substrate recognition pattern of these endolysins. The peptide-containing portion of peptidoglycan, NAM-L-alanyl-D-isoglutamine (MDP, muramyl dipeptide), was preferentially bound by CAP 1-1 and CAP 10-3. In contrast, the glycan backbone, represented by the NAG-NAM dimer, was more strongly bound by CAP 6-3, 7-1, and 12-3. This difference implies that the *C. acnes* phages may have two functional types of endolysins namely peptide-targeting and glycan-targeting. By targeting distinct structural elements of the bacterial cell wall, this specificity may indicate adaptability and improve overall lytic efficiency.

Key hydrogen-bonding residue analysis showed that some amino acids, especially histidine, are repeated in several CAP proteins, suggesting that they may play a part in ligand recognition. While unique residues in CAP 6-3, 7-1, and 12-3 probably contribute in selective NAG-NAM dimer binding, the conserved presence of ASP170 in CAP 1-1 and CAP 10-3 indicates a common mechanism for detecting MDP. These results offer mechanistic understanding of the molecular factors that may influence the substrate specificity of endolysin.

Interestingly, the preferential binding of CAP 1-1 and CAP 10-3 to MDP may also have immunological implications [34] . The bacterial peptidoglycan's minimal bioactive motif, muramyl dipeptide (MDP), is a known ligand of the innate immune receptor nucleotide-binding oligomerization domain-containing protein 2 (NOD2). Both the peptide and carbohydrate moieties of MDP are involved in binding at the leucine-rich repeat (LRR) domain of NOD2. NOD2-mediated signaling cascades are initiated by this contact, which activates NF-κB and produces pro-inflammatory cytokines. Crohn's disease susceptibility is closely correlated with NOD2 genetic variants that affect MDP recognition [35] . By sequestering free MDP, these endolysins (CAP 1-1 and CAP 10-3) could prevent its recognition by the intracellular receptor NOD2, thereby reducing NOD2-mediated inflammatory signaling. This mechanism could potentially lower inflammation associated with *C. acnes* infection. Thus, these endolysins could not only exert direct antibacterial effects but also modulate host immune responses, a feature that may be particularly relevant for therapeutic interventions targeting inflammatory skin conditions like acne.

The suggestion that MDP-binding endolysins might interfere with NOD2-mediated signaling should be considered hypothetical. While docking analyses indicate a potential for interaction with muramyl dipeptide (MDP), a known NOD2 ligand, these predictions cannot confirm whether such binding would occur under physiological conditions, nor whether it would meaningfully modulate host immune responses. Therefore, the proposed immunological role should be interpreted as a preliminary mechanistic hypothesis that requires validation through dedicated experiments, such as NOD2 reporter assays or cytokine-profiling studies.

Overall, our findings demonstrate the conserved and unique molecular characteristics of *C. acnes* phage endolysins, exposing the structural basis of their possible immunomodulatory functions as well as their substrate selectivity. This study is based entirely on computational analyses, including genomic annotation, domain architecture prediction, structural modeling, protein–ligand docking, and in silico interaction analysis. Although these approaches provide valuable preliminary insights into the potential functional diversity of endolysins, the biological relevance of the predicted catalytic preferences, binding patterns, and antibacterial roles cannot be fully confirmed without experimental validation.

Future studies will therefore focus on cloning and recombinant expression of selected endolysins, followed by biochemical assays to measure muralytic activity, substrate-binding affinity, and bactericidal efficacy against *C. acnes*. Additional experiments such as site-directed mutagenesis and glycan or peptide fraction–specific activity assays will be essential to verify the mechanistic hypothesis proposed in this work. Accordingly, the interpretations presented here should be considered predictive and hypothesis-generating until validated through laboratory experiments. Endolysin-based treatments have the potential to be a novel approach to treating acne along with reducing inflammation given their dual function. Future research should concentrate on experimental validation to confirm the substrate specificity, lytic activity and immunomodulatory effects of these endolysins *in vitro* and in vivo as these findings are solely based on in silico analysis.

## Conclusion

This study demonstrates computationally how *C. acnes* phage endolysins interact with typical peptidoglycan cell wall components of *C. acnes* such as NAM-L-alanyl-D-isoglutamine (MDP) and NAG-NAM dimers. According to our docking studies, the residue-level interactions that appear to govern substrate specificity across various endolysin variants are both conserved and unique. Importantly, some endolysins' preferential binding to MDP suggests the possibility that they may play a part in regulating host immune signaling by preventing NOD2 activation and reducing inflammation. These results collectively offer preliminary understanding of the dual antibacterial and immunomodulatory properties of endolysins from *C. acnes* phage. Although the present study offers detailed computational predictions of endolysin substrate specificity, domain-function relationships, and potential immunomodulatory interactions, molecular docking inherently lacks the ability to fully capture dynamic enzymatic processes, conformational flexibility, and the complexity of immune receptor engagement. Therefore, the mechanistic interpretations presented here remain predictive and require experimental confirmation. To verify the biological significance of these interactions and assess their viability as therapeutic agents, however, experimental validation like zymography and peptidoglycan cleavage assays to assess muralytic activity, LC-MS–based muropeptide analysis to determine substrate preference, and NOD2 or NF-κB luciferase reporter assays to evaluate immunological signaling is necessary as this work is limited to computational predictions. Incorporating these functional assays in follow-up studies will be essential to verify the catalytic roles, ligand-binding patterns, and immune-modulating potential suggested by our *in silico* results.

## Supplemental Materials

Supplementary data for this paper are available on-line only at http://jmb.or.kr.



## Figures and Tables

**Table 1:** Genomic characteristics of the isolated *C. acnes* phages.

Phage	Genome size (bp)	GC content (%)	CDSs
CAP 1-1	29534	54.03	40
CAP 1-2	29633	54.04	42
CAP 1-3	29655	54.02	42
CAP 2-1	29614	54.05	42
CAP 2-2	29614	54.04	42
CAP 6-3	29449	54.25	40
CAP 7-1	29983	53.61	45
CAP 7-2	29981	53.59	46
CAP 7-3	29943	53.60	46
CAP 9-2	29983	53.61	47
CAP 10-1	29656	54.03	42
CAP 10-3	29625	53.86	42
CAP 12-1	29676	54.0	42
CAP 12-2	29676	54.0	42
CAP 12-3	29676	53.97	44

**Table 2:** Binding affinities for protein-ligand, calculated using Autodock Vina, kcal/mol.

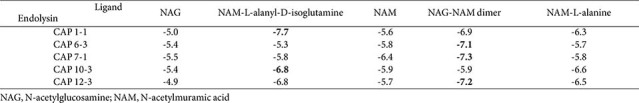

**Table 3:** Binding pocket residues of five selected endolysins.

Proteins	Best Ligand	Key interacting residues (H-bonds)
CAP 1-1	NAM-L-alanyl-D-isoglutamine	CYS:26, HIS:23,77,156,168, ASP:170
CAP 6-3	NAG-NAM dimer	GLU:88, HIS:23,156,168, ALA:24, SER:98
CAP 7-1	NAG-NAM dimer	TRP:77, GLY:54, ALA:25, GLU:89, HIS:169
CAP 10-3	NAM-L-alanyl-D-isoglutamine	THR:26,44, ALA:25, ASP:170, ARG:40
CAP 12-3	NAG-NAM dimer	TYR:47, GLU:89, SER:99, HIS:157,169

NAG, N-acetylglucosamine; NAM, N-acetylmuramic acid

## References

[1] Tan JKL, Bhate K (2015). A global perspective on the epidemiology of acne. Br. J. Dermatol..

[2] Skroza N, Tolino E, Mambrin A, Zuber S, Balduzzi V, Marchesiello A (2018). Adult acne versus adolescent acne: a retrospective study of 1,167 Patients. J. Clin. Aesthet.Dermatol..

[3] Hazarika N, Archana M (2016). The psychosocial impact of acne vulgaris. Indian J. Dermatol..

[4] Xu S, Zhu Y, Hu H, Liu X, Li L, Yang B (2021). The analysis of acne increasing suicide risk. Medicine.

[5] Cros MP, Mir-Pedrol J, Toloza L, Knödlseder N, Maruotti J, Zouboulis CC (2023). New insights into the role of *Cutibacterium acnes*-derived extracellular vesicles in inflammatory skin disorders. Sci. Rep..

[6] Alkhawaja E, Hammadi S, Abdelmalek M, Mahasneh N, Alkhawaja B, Abdelmalek SM (2020). Antibiotic resistant *Cutibacterium acnes* among acne patients in Jordan: a cross sectional study. BMC Dermatol..

[7] Zhang S, Chu M, Sun X (2025). The arms race in bacteria-phage interaction: deciphering bacteria defense and phage anti-defense mechanisms through metagenomics. Front. Microbiol..

[8] Niazi SK (2025). Bacteriophage therapy: discovery, development, and FDA approval pathways. Pharmaceuticals.

[9] Subramanian A (2024). Emerging roles of bacteriophage-based therapeutics in combating antibiotic resistance. Front. Microbiol..

[10] Pirnay JP, Djebara S, Steurs G, Griselain J, Cochez C, De Soir S (2024). Personalized bacteriophage therapy outcomes for 100 consecutive cases: a multicentre, multinational, retrospective observational study. Nat. Microbiol..

[11] Principi N, Silvestri E, Esposito S (2019). Advantages and limitations of bacteriophages for the treatment of bacterial infections. Front. Pharmacol..

[12] Liu H, Hu Z, Li M, Yang Y, Lu S, Rao X (2023). Therapeutic potential of bacteriophage endolysins for infections caused by Gram-positive bacteria. J. Biomed Sci..

[13] Vukov N, Moll I, Bläsi U, Scherer S, Loessner MJ (2003). Functional regulation of the *Listeria monocytogenes* bacteriophage A118 holin by an intragenic inhibitor lacking the first transmembrane domain. Mol. Microbiol..

[14] Wang IN, Smith DL, Young R (2000). HOLINS: the protein clocks of bacteriophage infections. Annu. Rev. Microbiol..

[15] Arakelian AG, Chuev GN, Mamedov TV (2024). Molecular docking of endolysins for studying peptidoglycan binding mechanism. Molecules.

[16] Meng XY, Zhang HX, Mezei M, Cui M (2011). Molecular docking: a powerful approach for structure-based drug discovery. Curr. Comput. Aided Drug Des..

[17] Trott O, Olson AJ (2010). AutoDock Vina: Improving the speed and accuracy of docking with a new scoring function, efficient optimization, and multithreading. J. Comput.Chem..

[18] Han M-H, Khan SA, Moon G-S (2023). *Cutibacterium acnes* KCTC 3314 growth reduction with the combined use of bacteriophage PAP 1-1 and Nisin. Antibiotics (Basel).

[19] Kim JI, Hasnain MA, Moon GS (2023). Expression of a recombinant endolysin from bacteriophage CAP 10-3 with lytic activity against *Cutibacterium acnes*. Sci. Rep..

[20] Bolger AM, Lohse M, Usadel B (2014). Trimmomatic: a flexible trimmer for Illumina sequence data. Bioinformatics.

[21] Li D, Liu CM, Luo R, Sadakane K, Lam TW (2015). MEGAHIT: an ultra-fast single-node solution for large and complex metagenomics assembly via succinct de Bruijn graph. Bioinformatics.

[22] Mikheenko A, Prjibelski A, Saveliev V, Antipov D, Gurevich A (2018). Versatile genome assembly evaluation with QUAST-LG. Bioinformatics.

[23] Manni M, Berkeley MR, Seppey M, Zdobnov EM (2021). BUSCO: assessing genomic data quality and beyond. Curr. Protoc.

[24] Lowe TM, Chan PP (2016). tRNAscan-SE On-line: integrating search and context for analysis of transfer RNA genes. Nucleic Acids Res..

[25] Lawal B, Kuo YC, Amos Onikanni S, Chen YF, Abdulrasheed-Adeleke T, Oluwaseun Fadaka A (2023). Computational identification of novel signature of T2DM-inducednephropathy and therapeutic bioactive compounds from *Azanza garckeana*. Am. J. Transl. Res..

[26] Schrodinger LLC (2015). The PyMOL molecular graphics system. Version.

[27] Cavallo I, Sivori F, Truglio M, De Maio F, Lucantoni F, Cardinali G (2022). Skin dysbiosis and *Cutibacterium acnes* biofilm in inflammatory acne lesions of adolescents. Sci. Rep..

[28] Beirne C, McCann E, McDowell A, Miliotis G (2022). Genetic determinants of antimicrobial resistance in three multi-drug resistant strains of *Cutibacterium acnes* isolated from patients with acne: a predictive in silico study. Access Microbiol..

[29] Ranveer SA, Dasriya V, Ahmad MF, Dhillon HS, Samtiya M, Shama E (2024). Positive and negative aspects of bacteriophages and their immense role in the food chain. NPJ Sci. Food.

[30] Marinelli LJ, Fitz-Gibbon S, Hayes C, Bowman C, Inkeles M, Loncaric A, *et al*. 2012. *Propionibacterium acnes* bacteriophages display limited genetic diversity and broad killing activity against bacterial skin isolates. *mBio* **3:** e00279-12. https://doi.org/10.1128/mbio.00279-12. 10.1128/mBio.00279-12 23015740 PMC3448167

[31] Sørensen PE, Van Den Broeck W, Kiil K, Jasinskyte D, Moodley A, Garmyn A (2020). New insights into the biodiversity of coliphages in the intestine of poultry. Sci. Rep..

[32] Almpanis A, Swain M, Gatherer D, McEwan N (2018). Correlation between bacterial G+C content, genome size and the G+C content of associated plasmids and bacteriophages. Microb. Genom..

[33] Brüggemann H, Lood R (2013). Bacteriophages infecting *Propionibacterium acnes*. Biomed Res. Int..

[34] Bahr GM, Chedid L (1986). Immunological activities of muramyl peptides. Fed. Proc..

[35] Lauro ML, D'Ambrosio EA, Bahnson BJ, Grimes CL (2017). Molecular recognition of muramyl dipeptide occurs in the leucine-rich repeat domain of Nod2. ACS Infect. Dis..

