# Supplementary material for: Substrate Specificity and Immunological Implications of Cutibacterium acnes Phage Endolysins
Source: J Microbiol Biotechnol. 2026 Jan 21;36:e2509038. doi: 10.4014/jmb.2509.09038 (PMC12861721; doi:10.4014/jmb.2509.09038)
Supplement: Supplementary file 1 [file jmb-36-e2509038-supple.pdf]

J. Microbiol. Biotechnol. 2026. 36: e2509038
https://doi.org/10.4014/jmb.2509.09038

Bacteriophages	Accession IDs
CAP 1-1	OP491959
CAP 1-2	PQ361272
CAP 1-3	PQ198863
CAP 2-1	PQ198865
CAP 2-2	PQ198867
CAP 6-3	OR178992.1
CAP 7-1	PQ198869
CAP 7-2	PQ198871
CAP 7-3	PQ198872
CAP 9-2	PQ198873
CAP 10-1	PQ198870
CAP 10-3	OR039357
CAP 12-1	PQ198868
CAP 12-2	PQ198866
CAP 12-3	PQ198864
